# Evaluation of Salivary HLA-DR4 and MMP-8 Levels Along with *Porphyromonas gingivalis* in Periodontitis Patients with Rheumatoid Arthritis

**DOI:** 10.12688/f1000research.164552.1

**Published:** 2025-06-23

**Authors:** Shaymaa Abdulkareem Nori, Batool Hassan Al-Ghurabi, Nik Nairan Abdullah

**Affiliations:** 1 Department of Basic Science, College of Dentistry, University of Baghdad, Baghdad, Baghdad Governorate, Iraq; 2 Department Public Health Medicine, Faculty Medicine, Universiti Teknologi, Sungai Buloh Campus, Selangor, Malaysia

**Keywords:** Periodontitis, Rheumatoid arthritis, Porphyromonas gingivalis, human leukocyte antigen-DR4, Matrix metaloprotienase-8.

## Abstract

**Background:**

Periodontitis (PD) is a persistent infectious inflammatory condition that affects the tissues supporting teeth and results in a progressive deterioration of the alveolar bone. Rheumatoid arthritis (RA), an autoimmune ailment that has been linked with raised periodontal disease severity. This study aimed to assess the clinical and immunological characteristics of PD in individuals with and without RA and to compare these characteristics with a healthy control group.

**Methods:**

The study included three groups; thirty patients with PD, thirty patients with PD and RA, and twenty healthy control participants. Clinical periodontal parameters [plaque Index (PLI), bleeding on probing (BOP), probing pocket depth (PPD), and clinical attachment loss (CAL)] were assessed. Salivary biomarkers, including human leukocyte antigen-DR4 (HLA-DR4), matrix metalloproteinase-8 (MMP-8), and anti-citrullinated protein antibody (ACPA), were analyzed by enzyme-linked immunosorbent assay (ELISA), and the microbial load of *Porphyromonas gingivalis* ( *P. gingivalis*) was determined using quantitative real time polymerase chain reaction (qRT-PCR).

**Results:**

The current findings showed that salivary level of HLA-DR4 was decreased in patients’ groups as compared to healthy control group. Whilst, salivary levels of MMP-8 and ACPA were elevated in patients’ groups in comparison to control group, with no substantial differences found across both patient groups. On the other hand, microbial load was significantly higher in the PD with and without RA groups than that in controls, with a positive correlation between microbial load and CAL in the PD-RA group.

## Introduction

Periodontitis is a profound and irreversible condition affecting the periodontium, marked by a complex interplay of infections that incite inflammation, ultimately resulting in the loss of vital supporting connective tissue and alveolar bone ( Mahmood and Al-Ghurabi, 2020, Domokos et al., 2024 and Al-Daragi et al., 2024). Severe PD is considered as the main cause of edentulism among adults, affecting about 11% of the global adult demographics ( Kwon et al., 2021 and Daily et al., 2023a). RA is a persistent, widespread inflammatory and autoimmune condition presenting considerable obstacles owing to its relentless nature, resulting in practical impairment along with premature fatality. It influences around 1% of the worldwide population, with higher occurrence rates noted among women and the elderly ( Kang et al., 2024 and Abdullah et al., 2024). Epidemiological studies investigating the connection between PD and RA present a spectrum of findings, showcasing a diverse prevalence of PD in RA patients, ranging from 28% to 85%, with 11-14% diagnosed with severe PD ( Punceviciene et al., 2021). PD and RA exhibit parallels in their pathophysiological progression, immunological regulation, hereditary susceptibility, infiltration of inflammatory cells, and the involvement of enzymes and cytokines in immunological reactions ( Nguyen et al., 2020 and Nori and Al-Ghurabi, 2025). *P. gingivalis,* a pathogenic bacterium associated with periodontal disease, is the sole inhabitant of the oral cavity possessing the unique enzyme that transforms arginine into citrulline. It has been suggested that upon infection with *P. gingivalis*, a periodontal tissue protein undergoes citrullination by this enzyme, leading to the formation of ACPA, which then creates an immune complex with citrullinated proteins in the joints, consequently triggering RA ( Mauramo et al., 2021).

The connection between PD and RA is further underscored by a specific genetic predisposition and associated environmental risk factors, such as smoking. Both conditions exhibit a shared genetic profile, particularly the shared epitope HLA-DRB1 alleles that code for HLA-DR4. Additionally, polymorphisms in genes that encode inflammatory cytokines, along with interleukin-1 combined risk alleles, may create a synergistic effect on bone destruction in joints and the periodontium, potentially increasing susceptibility to both RA and PD ( Naaom et al., 2017, Sorsa et al., 2020 and Ancuta et al., 2017). Furthermore, the alleles of HLA-DRB1 that encode class II major histocompatibility complex’s beta chain possess the ability to bind citrullinated peptides, potentially enhancing the auto-antigenic citrullinated peptide’s immunogenicity associated with RA ( de Molon et al., 2019). Sandal et al. revealed a promising association of HLA-DRB1 in the production of ACPA following *P. gingivalis* oral infection in a mouse model, suggesting a potential causal relationship between PD and RA ( Sandal et al., 2016).

A multitude of inflammatory cells release MMP enzymes. In PD and RA, genetically separate but structurally similar MMPs destroy almost all extracellular matrix constituents. Increased concentrations of MMP-8 have been linked to PD and expedited disease development ( Mauramo et al., 2021). It is significant that specifically MMP-8 has been identified as an effective biomarker for PD. Besides PD, new research indicates that MMP-8 is linked to chronic inflammation and inflammatory conditions such as RA and cardiovascular illnesses ( Raheem & Ahmed, 2014, Äyräväinen et al., 2018). Interestingly, there is no study has investigated the connection among the salivary levels of HLA-DR4, MMP-8, ACPA and *P. gingivalis* altogether in PD subjects with and without RA. Thus, this illuminates the current study, which seeks to assess the accuracy of salivary HLA-DR4, MMP-8, ACPA and *P. gingivalis* levels in differentiating patients with PD with and without RA from clinically healthy individuals, and to correlate these biomarkers with the periodontal parameters, for better understanding the immune response and inflammation mechanisms involved in the development and progression of both diseases.

## Methods

### Study design

It is a case-control research which relies on observational data. This research was conducted within the College of Dentistry/University of Baghdad, spanning the period from 15 October 2024 to 15 January 2025. Ethical considerations were paramount in the present study, guided via the World Medical Association’s Helsinki Declaration, and ethical approval was obtained from the ethical committee at the Dentistry College/University of Baghdad (Reference Number: 940, Project number: 940824, Date: 14-10-2024).

### Research instrument

Three ELISA kits were used in this study for the analysis of salivary biomarkers. These kits include: HLA-DR4 ELISA kit (AFG™ Scientific company / USA), MMP-8 and ACPA ELISA kits (SunLong Biotech™ company/China). For *P. gingivalis* analysis, a bacterial DNA isolation kit (Presto™ Mini gDNA Bacteria Kit, Geneaid Biotech Ltd/Taiwan) was used, primers for *P. gingivalis* (Macrogen™ /South Korea), and qRT-PCR Master Mix (Luna ^®^ Universal/New England Biolabs/USA).

### Catalogue numbers

MMP-8 ELISA Kit = SL1156

ACPA ELISA Kit = SL2976HU

HLA-DR4 ELISA Kit = EK716715

Presto™ Mini gDNA Bacteria Kit = GBB100/101

Luna ^®^ Universal qPCR Master Mix = NEB #E3005S/L

### Study population

This study included 80 subjects aged between 30 and 55 years. All participants had a body mass index <25 kg/m ^2^. Diagnosis of the PD patients was established according to the classification criteria of periodontal diseases by Tonetti ( Tonetti et al., 2018). While RA diagnoses was established according to the American College of Rheumatology/European Alliance of Associations for Rheumatology 2010 criteria ( Aletaha et al., 2010). The chosen participants were categorised into:

• 20 healthy control participants with pristine periodontium.
• 30 participant suffering from generalized unstable PD with RA who had previously been treated with non-biological Disease-modifying anti-rheumatic drugs (PPD ≥4 mm, CAL ≥4 mm including at least 30% of teeth).
• 30 participant suffering from generalized unstable PD without RA (PPD ≥4 mm, CAL ≥4 mm including at least 30% of teeth).

### Inclusion criteria

The inclusion criteria were:

1. Generalized unstable PD patients with RA.
2. Generalized unstable PD patients without RA.
3. Age range between (30-55) years.
4. The existence of a minimum 20 natural teeth or more.
5. Subjects had PPD ≥4 mm, CAL ≥4 mm including at least 30% of teeth.

### Exclusion criteria

The exclusion criteria were:

1. Presence of systemic disorders other than RA, such as hypertension, thyroid diseases and diabetics.
2. Previous periodontal therapy for the last 6 months.
3. Pregnant and menopause women.
4. The use of antibiotics and/or anti-inflammatory medication in the last 3 months.
5. History of smoking or alcohol drinking.

### Sample size

Utilizing G power 3.1.9.7 (Program written by Franz-Faull, University of Kiel, Germany), the sample size was calculated with a power of 90% for the study, a two-sided alpha error of probability of 0.05, an effect size of F is 0.4 (large effect size), three groups, under these circumstances the sample size is approximately 80 participants. Effect size F is: Small = 0.1, medium = 0.25, large = 0.4 ( Cohen, 2013).

### Disease activity score-28

Each candidate in PD patients’ group with RA scored for disease activity, according to Disease activity score-28 **(**DAS-28). Rheumatologist specialists conducted the evaluation of disease activity using DAS-28 which is a weight multidimensional index that utilized by a medical expert for joint assessment, the patient’s subjective evaluation of their illness alongside the laboratory markers of inflammation, C-reactive protein (CRP), rheumatoid factor, ACPA and erythrocyte sedimentation rate (ESR) ( Van der Heijde et al., 1993). DAS-28 is an indicator akin to the original DAS, comprising of a 28 tender joint count (TJC), a 28 swollen joint count (SJC), ESR, along with a patient global assessment (PGA) on a visual analog scale (range, 0–100) ( Prevoo et al., 1995). The degree of disease activity might be expressed as low (DAS-28 ≤ 3.2), moderate (3.2 ≤ DAS-28 ≤ 5.1) or high (DAS-28 > 5.1). Using ESR, the preceding equation was used to compute the DAS-28 ( Van Gestel et al., 1998).

$$\mathrm{DAS}-28=\left(0.56\times {}^{`}\surd \mathrm{TJC}\right)+\left(0.28\times \surd \mathrm{SJC}\right)+\left(0.70\times \text{logn}\;\mathrm{ESR}\right)+\left(0.014\times \mathrm{PGA}\right)$$

### Saliva samples

Three ml of whole un-stimulated saliva was collected from study groups in a sterile cup. Subjects were asked to refrain from drinking and eating one hour before donation of saliva. Within one hour after collection, saliva centrifuged at 1000 × g for 15 minutes to eliminate debris and cellular matter, the supernatants were aspirated immediately, divided into three aliquots and kept at ≤-20°C until used ( Costa et al., 2021).

### Plaque samples

Subgingival plaque samples were collected using fine sterile Gracey curettes from the gingival sulcus in the control group and the four deepest periodontal pockets in the patient groups, placed immediately in eppendorf tube containing 0.5 ml TE buffer (10 mM TrisHCl, 1 mM EDTA, pH 7.6) and stored at (-40°C) ( Borges et al., 2009).

### Clinical periodontal parameters

Once saliva and plaque samples were collected, clinical parameters (PLI, BOP, PPD, and CAL) were assessed utilizing William’s periodontal probe. Each tooth was meticulously examined across six unique areas (Mesiobuccal, Buccal, Distobuccal, Mesiolingual, Lingual, and Distolingual) to assess BOP, PPD and CAL. Meanwhile, PLI scores were elegantly documented by scrutinizing just four surfaces (mesial, distal, labial/buccal, lingual/palatal). Third molar was omitted from all parameters assessment, with the exception of PLI. PLI was meticulously measured utilizing disclosing agents to ascertain dental plaque existence or non-existence ( O’Leary et al., 1972), while BOP percentage was recorded as 1 present 0 absent ( Mitani et al., 2024). The measurement separating the unrestricted gingival border to the pocket’s bottom is termed PPD. In contrast, CAL refers to the measurement from cemento-enamel junction (CEJ) to the pocket’s bottom in case of gingival regression. When the gingival margin is at the CEJ, both CAL and PPD are equal. At recession, the CAL is calculated by summing the extent of the recession along with PPD. If the gingival border sits above the CEJ, the CAL is determined by subtracting the measurement from the gingival border to the CEJ from the PPD ( Tonetti et al., 2018).

### Detection of HLA-DR4, MMP-8 and ACPA in saliva

Using ELISA technology, salivary biomarkers, HLA-DR4, MMP-8 and ACPA levels were assessed for each subject. All ELISA kits used sandwich- ELISA technique and the optical density is spectrophotometrically measured at a wave length of 450 nm. Optical density exhibited a direct proportionality to salivary biomarkers concentration.

### Reagent preparation

Twenty ml concentrated wash buffer was combined with 580 ml deionized water. Then by using a standard pipette 50 ul, standard diluent was diluted within every single tube. 100 ul standard was pipetted within the fifth tube. Moreover, 100 ul was transferred from the fifth to the fourth tube. 50 ul was pipetted from the fourth tube to the third tube to create dilution series. The pure Standard served as the high standard. Sample Diluent served as the zero standard blank well.

### Estimation of salivary HLA-DR4

One hundred μl standard diluent was pipetted into new micro-centrifuge tubes (labeled 5-0). 200 ul of standard was pipetted into tube 5, vortex. 200 μl of tube 5 was transferred to tube 4, then 100 μl of tube 4 was transferred to tube 3, repeated vortexing to tube 1. Tube 0 was just sample diluent. After that 50 μl of standards was added in replicate to provide 96-well plate.

Utilizing a multichannel pipette, 40 μl of sample diluent was added to all wells that contain samples. Next, 10 μl samples was added to wells in replicate, then the wells were covered and incubated at 37°C for 40 minutes. After the incubation, 300 μl wash solution/wash was added and the plate was washed 5 times. Further, 50 μl HRP was added to all wells excluding standard tube 0, then covered and incubated 40 minutes at 37°C.

Moreover, 300 μl wash solution/wash was added and the plate was washed 5 times. Subsequently, 50 μl of chromogen A was added to all wells followed by 50 μl of chromogen B. Light was avoided while preforming this step. The wells were covered and incubated at 37°C for 20 minutes. Eventually, 50 μl of stop solution was added to each well and read immediately at 450 nm.

The plate’s analysis was conducted by subtracting the blank well’s measurement from every other absorbances. In order to get the actual concentration, the concentration was multiplied by five since the samples were first diluted five times.

### Estimation of salivary MMP-8

The standard was diluted by small tubes first, pipetted the volume of 50 μl from each tube to microplate well. In the micro-ELISA strip plate, a well empty was left as blank control. In sample wells, 40 μl Sample dilution buffer and 10 μl sample were added. Samples were placed inside the bottom without contacting the well wall and blended thoroughly with a light shake. Subsequently, the sample had to incubate for 30 minutes at 37°C after being capped with a closure plate membrane.

After carefully peeling off the membrane of the closure plate, aspirating the contents, and refilling it with the wash solution, the concentrated washing buffer was diluted with distilled water 30 times for 96T and 20 times for 48T. After letting the wash solution sit for 30 seconds, it was flushed and the process was repeated five times.

Each well, with the exception of the blank control well, received fifty μl of HRP-conjugate reagent, and the incubation process was carried out in the same manner as stated before. The act of washing was carried out, as was described sooner.

Fifty μl of Chromogen Solution A and 50 μl of Chromogen Solution B have been placed to each well, merged gently, and incubated at 37°C for 15 minutes. Light exposure was avoided during the colouring process.

Finally 50 μl stop solution was added to each well to terminate the reaction. The color in the well changed from blue to yellow.

A microtiter plate reader was used to measure the OD at 450 nm. The OD of the control well was set as zero. After applying the stop solution, the assay was conducted no more than 15 minutes later.

### Assay procedure and estimation of salivary ACPA

All steps of the procedure were similar to those previously mentioned in the measurement of MMP-8.

### Detection of *Pophyromonas gingivalis* in dental plaque

Using qRT-PCR technology, the microbial load of *p. gingivalis* in subgingival dental plaque was determined for each participant. Genomic DNA was isolated from dental plaque samples. For specific detection and quantification of *P. gingivalis* bacteria, primers were synthesized. Then target bacterial DNA sequences were amplified using qPCR, with fluorescence monitored in real-time during each amplification cycle. The cycle threshold, defined as the cycle number at which fluorescence exceeds a predetermined threshold, was recorded for each sample. Quantification was achieved by comparing the cycle threshold values of the samples to a standard curve generated from serial dilutions of known DNA concentrations ( Kareem et al., 2022).

### Statistical analysis

All statistical analyzes of the data were performed and processed with the computerized analysis statistical package for the social sciences (SPSS) software program (version 25, IBM, USA) and GraphPad Prism software (version 9.0). Statistical variance was deemed significant when p < 0.05. The data were presented as descriptive statistics involving mean and standard deviation. Clinical and immunological data distribution was determined by Shapiro Wilk test. Inferential statistics were used to accept or reject the statistical hypotheses which included: Chi-square test and one-way analysis of variance (ANOVA) parametric test were implemented to record the variations across a minimum of three distinct groups, Tukey honestly significant difference (HSD)/post hoc test was utilized to ascertain the statistical significance of the relationship between two sets of data. For non-parametric data Kruskal-Wallis test and post-hoc Dunn’s test was used to test the statistical difference between groups. Spearman correlation coefficient test was used to determine the correlation among different parameters. In addition, to show the diagnostic potential of cytokines a receiver operating characteristic (ROC) curve was established.

## Results

**Figure 1.**
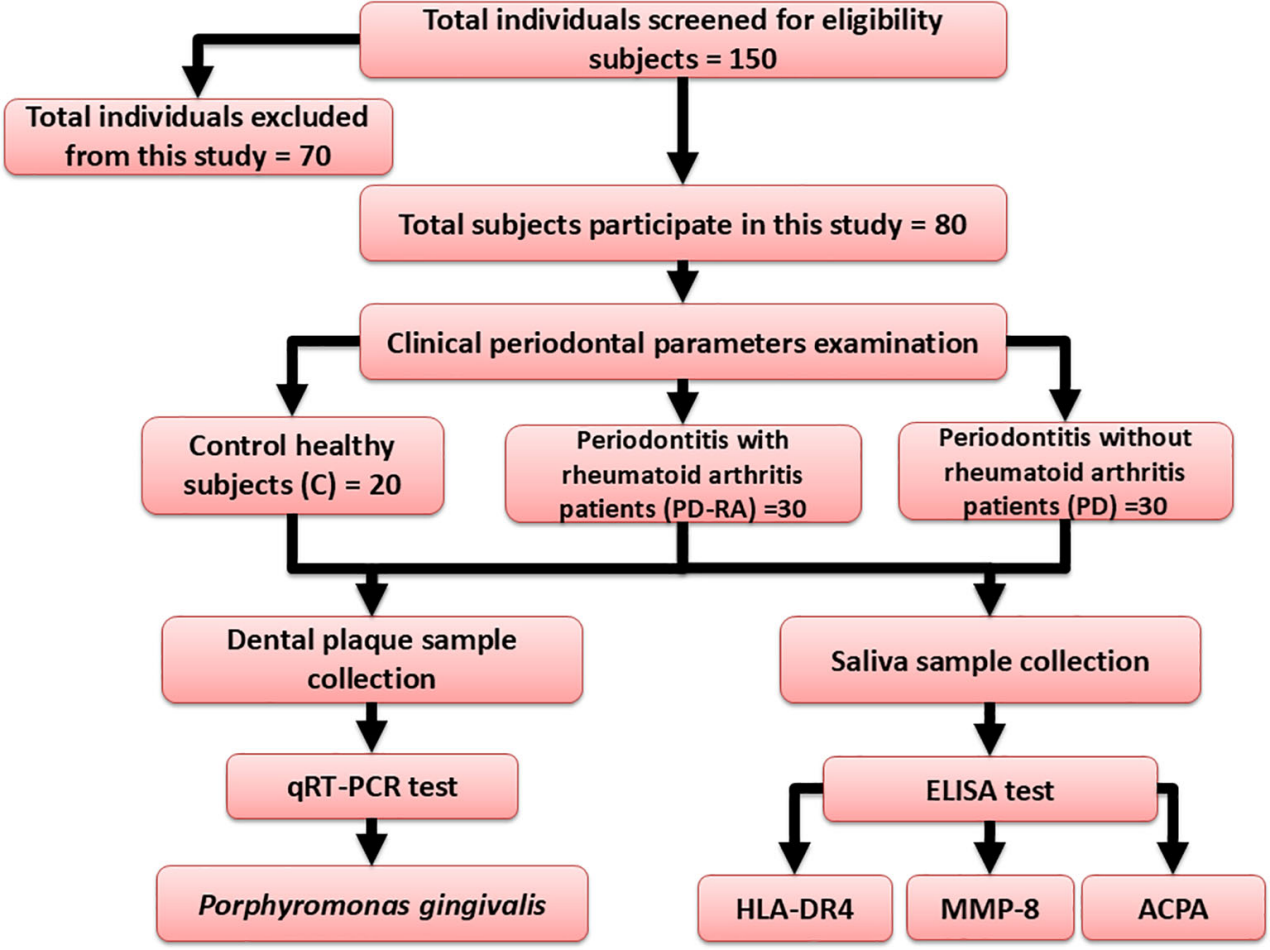
Flow chart of the study design.

After implementing the inclusion and exclusion criteria, 70 individuals were excluded from the study, leaving 150 subjects to be evaluated for recruiting eligibility. Μltimately, only 30 male and female PD with RA patients, 30 male and female PD patients and 20 control participants were incorporated into the study ( Figure 1).

### Demographic characteristics of the study participants

The results of the present study revealed no significant differences in the age and sex of the participants between all groups. Ratio male/female between patients and healthy control group was 1:1.4. While, the mean disease duration in the RA group was 7.0 ± 1.35 years and the mean DAS-28 was 6.89 ± 1.38. Moreover, all periodontal parameters, PLI, BOP, PPD and CAL, were statistically greater for the PD groups with and without RA, compared to the healthy periodontium participants ( *p *< 0.05), ( Table 1).

**Table 1.** Demographic and clinical characteristics of the study groups. Mean percentage and values of periodontal parameters in study groups.

Demographic characteristics	Study groups			*P*- value
	PD with RA n = 30	PD n = 30	Healthy control n = 20	
Age (years)				0.126 ^NS^
Mean ± SD	44.90 ± 7.20	45.33 ± 8.04	41.11 ± 7.70	
Disease duration of RA				
Mean ± SD	7.0 ± 1.35	-	-	-
DAS-28				
Mean ± SD	6.89 ± 1.38	-	-	-

Study groups	Sex		* P*- value
	Male N(%)	Female N(%)	
PD n = 60	25 (41.6%)	35 (58.4%)	0.801 ^NS^
Healthy control n = 20	7 (35%)	13 (65%)	
Ratio male/female = 1:1.4			

Periodontal parameters Mean ± SD	Study groups			* P*- value
	PD with RA n = 30	PD n = 30	Healthy control n = 20	
PLI	68.16 ± 10.66	59.53 ± 16.97	10.95 ± 4.47	0.0000 ^*^
BOP	72 ± 10.12	54 ± 11.8	4.9 ± 1.20	0.0000 ^*^
PPD	6.45 ± 0.89	6.19 ± 0.99	_	1.452 ^NS^
CAL	5.86 ± 1.31	5.83 ± 1.41	_	0.470 ^NS^

NS: Non–Significant, SD: Standard Deviation, No.: Number, %: Percentage

### Immunological salivary biomarkers analysis

The current study noticed a significant decrease of salivary HLA-DR4 (P ˂ 0.05) in patient groups as compared with control group. Nevertheless, the findings indicated that there was no statistically significant difference (p > 0.05) in the salivary HLA-DR4 concentration between PD with and without RA groups, as shown in ( Table 2). The mean rank levels of salivary MMP-8 and ACPA in patients’ groups were significantly higher than control group, whereas, intergroup comparisons of mean values of MMP8 and ACPA between PD with and without RA groups indicated that there was no statistically significant difference (p > 0.05), as shown in ( Table 2).

**Table 2.** The disparity in the average rank values of salivary biomarkers (ng\L) and microbial load in study groups.

Immunological biomarkers and microbial load	Study groups				P value
	PD with RA n = 30	PD n = 30	Healthy control n = 20	Kruskal Wallis Test	
HLA-DR4Mean rank	38.12 ^a^ *	30.85 ^b^ ^NS^	58.55 ^c^ *	17.56	0.000 ^*^
ACPA Mean rank	50.7 ^a^ *	43.93 ^b^ ^NS^	20.05 ^c^ *	21.92	0.001 ^*^
MMP-8 Mean rank	44.58 ^a^ *	44.2 ^b^ ^NS^	28.82 ^c^ *	6.74	0.034 ^*^
*P. gingivalis* Mean rank	51.47 ^a^ *	55.40 ^b^ ^NS^	20.58 ^c^ *	31.78	0.001 ^*^

^a^ Comparison between PD with RA and healthy control group;

^b^ Comparison between PD with and without RA groups;

^c^ Comparison between PD without RA and healthy control group; NS: not significant;

* Significant.

### Genetic analysis of Pophyromonas gingivalis

The comparison of the mean rank values of *P. gingivalis* microbial load in study groups showed that there was statistically significant difference between the patient groups and the control group. However, Intergroup comparisons of mean values of *P. gingivalis* microbial load between PD with and without RA groups indicated that there was no statistically significant difference (p > 0.05), as shown in ( Table 2).

### Correlation between salivary biomarkers and microbial load with clinical periodontal parameters

A substantial correlation (p = 0.018) existed in PD without RA group between HLA-DR4 and CAL. Moreover, A notable connection (p = 0.025) existed in PD with RA group between *P. gingivalis* concentration and CAL ( Table 3).

**Table 3.** Correlation between biomarkers and clinical periodontal parameters in periodontitis subjects with and without rheumatoid arthritis.

Groups	Periodontal parameters	ACPA	HLA-DR4	MMP8	Microbial load
PD with RA	PLI	r = 0.064	r = 0.009	r = 0.119	r = 0.241
		p = 0.736	p = 0.599	p = 0.528	p = 0.197
	BOP	r = 0.239	r = 0.141	r = 0.176	r = 0.192
		p = 0.201	p = 0.456	p = 0.350	p = 0.307
	PPD	r = 0.186	r = 0.186	r = 0.353	r = 0.220
		p = 0.325	p = 0.309	p = 0.054	p = 0.241
	CAL	r = 0.282	r = 0.139	r = 0.075	r = 0.406
		p = 0.129	p = 0.463	p = 0.692	p = 0.025
PD	PLI	r = 0.073	r = 0.133	r = 0.212	r = 0.191
		p = 0.701	p = 0.481	p = 0.260	p = 0.310
	BOP	r = 0.347	r = 0.017	r = 0.225	r = 0.059
		p = 0.060	p = 0.926	p = 0.231	p = 0.756
	PPD	r = 0.176	r = 0.055	r = 0.223	r = 0.162
		p = 0.351	p = 0.769	p = 0.234	p = 0.389
	CAL	r = 0.063	r = 0.425	r = 0.083	r = 0.162
		p = 0.738	p = 0.018	p = 0.662	p = 0.387

### Diagnostic accuracy of salivary biomarkers

**Figure 2.**
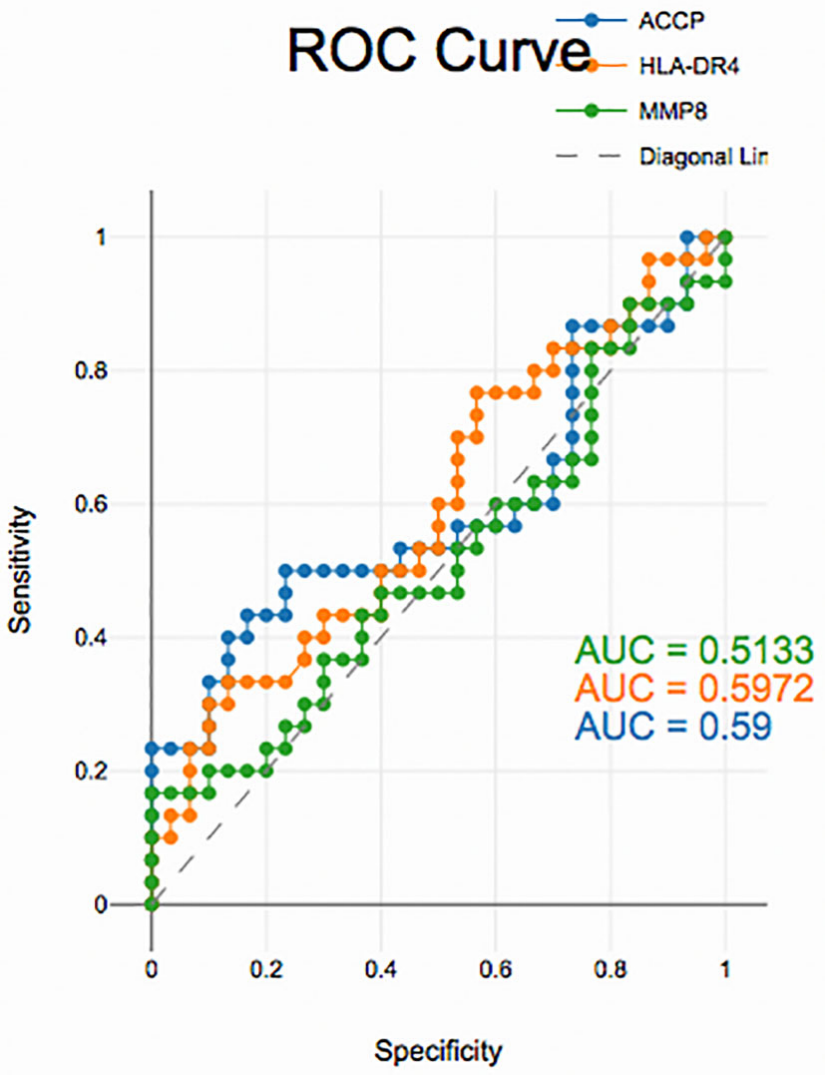
Receiver operating curves for salivary biomarkers in PD with RA vs. PD without RA.

**Figure 3.**
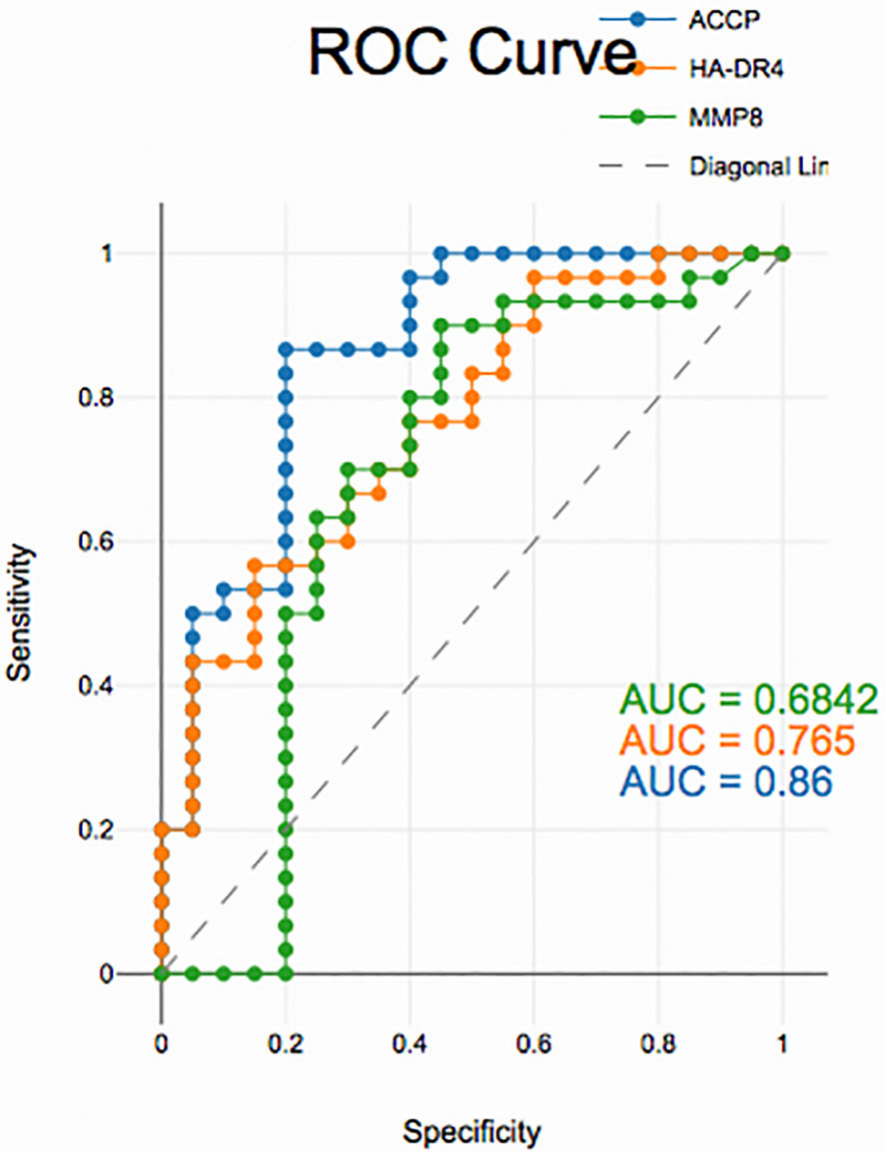
Receiver operating curves for salivary biomarkers in PD with RA vs. control

**Figure 4.**
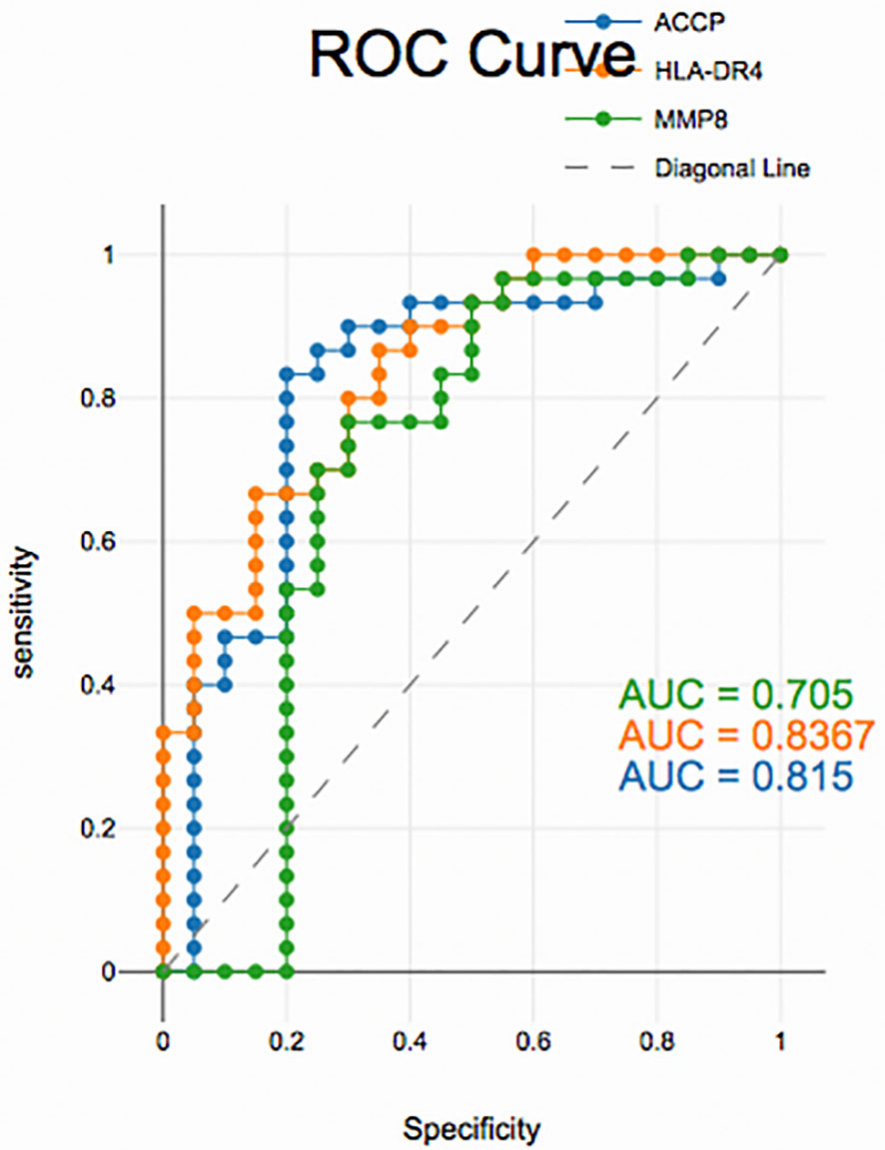
Receiver operating curves for salivary biomarkers in PD without RA vs. control.

PD with and without RA according to ROC data had good accuracy in assessing HLA-DR4, MMP-8 and ACPA sensitivity and specificity for distinguishing PD from healthy periodontium ( Figure 2), ( Figure 3) and ( Figure 4).

### Correlation of *Pophyromonas gingivalis* microbial load counts with salivary biomarkers in Periodontitis patients with and without Rheumatoid Arthritis

Spearman correlation test coefficients (r) calculated between *P.gingivalis* microbial load counts with salivary biomarkers in PD subjects with and without RA. The results demonstrated significant positive correlations between MMP-8 with both *P.gingivalis* microbial load counts, (r = 0.540_ p = 0.002) and ACPA (r = -0.359_ p = 0.050) in PD with RA group. While in PD group the results revealed significant positive correlation between MMP-8 and ACPA only, (r = 0.433_ p = 0.016).

## Discussion

The clinical periodontal assessments revealed significant differences in PLI and BOP between both PD with and without RA groups in comparison to control group, indicating increased periodontal inflammation and bacterial presence in these groups. PD with RA group had superior PLI and BOP values than the PD group, suggesting that RA exacerbates periodontal inflammation. This aligns with previous studies ( Daily et al., 2023b, Kim et al., 2018, Rodríguez-Lozano et al., 2019 and Hamed & Ali, 2017) showing higher periodontal indices in RA patients, indicating that RA may worsen periodontal inflammation. However, no significant differences in PPD and CAL were found between PD with and without RA groups, which was analogous to the findings of a study done by Varshney et al ( Varshney et al., 2021) The outcomes of the current study propose that RA influences the inflammatory processes in periodontal disease, leading to more pronounced inflammation but not necessarily increased tissue destruction in the short term.

Salivary biomarkers were analyzed to investigate potential immunological factors contributing to periodontal disease progression. Among the biomarkers, HLA-DR4 levels were significantly lower in PD with and without RA groups in comparison to controls, though no substantial disparity was noticed across PD with RA and PD without RA groups. However, studies investigating the role of HLA-DR4 in periodontal disease among RA patients have yielded mixed results. Roudier suggested that HLA-DR4 is prevalent in Caucasian RA patients ( Roudier, 2006), while there was no statistical significance in the prevalence of DR4 in RA patients versus controls within the Iranian population ( Salesi et al., 2016). Another study conducted in Sudan reported that HLA-DRB1*07, HLA-DQB1*02, and *06, which are HLA-DR4 alleles and haplotypes, were significantly higher in healthy control group and showed protection against RA ( Ali et al., 2023). As for HLA-DR4 association with PD, Bonfil et al. reported that a higher frequency of at least one of the alleles compared to healthy persons: DRB1*0401, DRB1*0404, DRB1*0405, or DRB1*0408 ( Bonfil et al., 1999). Conversely, research including Japanese patients indicated a higher prevalence of the DRB1*1401, DRB1*1501, DQB1*0503, DQB1*0602 alleles and infrequent occurrence of the DRB1*0405, DQB1*0401 alleles in early-onset PD patients than in controls ( Ohyama et al., 1996). One can not claim that the current study does coincide with the above motioned studies, since it investigated HLA-DR4 as a protein without analyzing its allelic variants. According to Gung et al., the expression of HLA-DR is positively correlated with interleukin IL-23 ( Jung et al., 2018). Interestingly, Sadeghi et al., reported that IL-23 level is usually higher in healthy control individuals than in PD ( Sadeghi et al., 2018). Furthermore, Patients taking methotrexate therapy usually show decreased levels of IL-23 as reported by Elghandour et al., ( Elghandour et al., 2013). The above mentioned studies support the significant decrease in HLA-DR4 level in PD with and without RA groups in comparison to control group. Furthermore, the current study found positive significant correlation between HLA-DR4 and CAL in PD without RA group. this finding is consistent with Bonfil et al., study which indicated a significant relationship between HLA-DR4 and CAL in severe and rapidly progressive PD ( Bonfil et al., 1999).

Regarding MMP-8 and ACPA, the results revealed significant differences between the patient groups compared to the healthy control group. Although no significant difference was found between the PD with and without RA groups, Eriksson et al., did not report significant differences in salivary MMP-8 or ACPA levels between PD with and without RA groups which support our findings ( Eriksson et al., 2022). While both conditions share similar immunological biomarkers, the presence of RA did not appear to alter the salivary concentrations of these markers. These findings could imply that the presence of RA may not contribute uniquely to MMP-8 and ACPA levels in saliva, which could be important for understanding the shared pathophysiological mechanisms between RA and PD.

In the analysis of *P. gingivalis* microbial load, the study found significant differences between the patient groups and healthy controls although no notable variations were seen across the PD with and without RA groups. Furthermore, the correlation between *P. gingivalis* microbial load and CAL in PD with RA group, but not in PD group, may suggest that *P. gingivalis* is significantly involved in the destruction of periodontal tissues among RA individuals, probably attributable to an altered immune reaction in these patients. This result coincides with Selimov et al., ( Selimov et al., 2020), Chen et al., ( Chen et al., 2023), Li et al., ( Li et al., 2022), Moura et al., ( Moura et al., 2021), and Fiorillo et al., ( Fiorillo et al., 2019) studies which highlighted the role of *P. gingivalis* in PD and RA considering it as a risk factor.

The significant positive correlation between MMP-8 and *P. gingivalis* microbial load in the PD with RA group suggests that the presence of RA may influence the inflammatory response to *P. gingivalis*, resulting in higher MMP-8 levels in the saliva. MMP-8 is an important biomarker of tissue degradation, particularly in periodontal tissues, and its elevation can be indicative of ongoing periodontal destruction. In the context of RA, which is characterized by chronic systemic inflammation, the immune system may be more responsive to *P. gingivalis*, leading to higher MMP-8 levels. Additionally, the correlation between MMP-8 and ACPA in the PD with RA group suggests that RA-related immune processes, including the production of ACPA, might further exacerbate periodontal inflammation and tissue breakdown, possibly contributing to the severity of periodontal disease in these patients.

On the other hand, in PD without RA group, the data revealed a significant positive correlation only between MMP-8 and ACPA, but not with *P. gingivalis* microbial load. This implies that while periodontal disease leads to increased levels of MMP-8, the microbial load of *P. gingivalis* may not be the sole driver of MMP-8 elevation in the absence of RA. Instead, the immune response reflected by elevated ACPA levels in PD patients could be playing a more prominent role in periodontal inflammation and tissue breakdown in this group.

Kuula et al., found that infections with *P. gingivalis* have been shown to influence the expression of MMP-8, suggesting a relationship between microbial load and MMP-8 levels during PD ( Kuula et al., 2009). In addition, Sorsa et al., reported that elevated MMP-8 levels have been associated with increased periodontal destruction, which may correlate with ACPA antibodies commonly found in RA individuals ( Sorsa et al., 2017).

The diagnostic analysis of salivary biomarkers, including ACPA, HLA-DR4 and MMP-8, using ROC curves demonstrated that these biomarkers had good sensitivity and specificity in differentiating PD from controls, yet, unreliable in distinguishing between PD with and without RA groups. This suggests that these biomarkers while useful in detecting PD may not be able to differentiate PD related to RA from PD alone. RA patients may have underlying systemic inflammation, which could affect the salivary biomarkers, but the biomarkers may not be specific enough to indicate whether PD is linked to RA. A limitation of the present research is the case-control design is observational and does not allow for establishing causality. It can show associations but not direct cause-and-effect relationships between PD and RA or between the biomarkers and disease outcomes. Although the study targeted 80 participants, the sample size is relatively small. A larger sample size could have provided more statistical power, improving the generalizability of the findings, especially when trying to differentiate between PD with and without RA.

## Conclusions

In conclusion, this research offers valuable perspectives on the intricate connection between PD and RA. While both PD with and without RA patients showed significant increases in periodontal parameters such as PLI and BOP, the presence of RA did not significantly alter the clinical severity of periodontal disease in terms of PPD and CAL. Immunological markers such as MMP-8, ACPA and *P. gingivalis* microbial load were elevated in both patient groups, with limited differences observed between PD with and without RA groups, suggesting that these biomarkers may reflect general periodontal inflammation rather than being specific to RA. The findings emphasize the need for further studies to explore the immune mechanisms linking RA and PD, as well as assessing the potential of salivary biomarkers as diagnostic tools for both conditions.

## Ethical approval

The research adhered to the Declaration of Helsinki and received approval from the Ethics Committee at the University of Baghdad, College of Dentistry, Iraq (number 940824, 14-10-2024).

## Consent to participate

All patients involved in this study provided their written informed consent.

## Data Availability

Zenodo: Patient raw data for paper (Evaluation of Salivary HLA-DR4 and MMP-8 Levels Along with Porphyromonas gingivalis in Periodontitis Patients with Rheumatoid Arthritis), https://doi.org/10.5281/zenodo.15376986 ( Nori et al., 2025a). Data is available under Creative Commons Zero v1.0 Universal 1. Zenodo: Case sheet (Evaluation of Salivary HLA-DR4 and MMP-8 Levels Along with Porphyromonas gingivalis in Periodontitis Patients with Rheumatoid Arthritis), https://doi.org/10.5281/zenodo.15376996 ( Nori et al., 2025b). Data is available under Creative Commons Zero v1.0 Universal
2. Zenodo: **Study photo**: https://doi.org/10.5281/zenodo.15377001 ( Nori et al., 2025c). Data is available under Creative Commons Zero v1.0 Universal
