# Supplementary material for: Evaluation of Salivary HLA-DR4 and MMP-8 Levels Along with Porphyromonas gingivalis in Periodontitis Patients with Rheumatoid Arthritis
Source: F1000Res. 2025 Oct 30;14:615. Originally published 2025 Jun 23. [Version 3] doi: 10.12688/f1000research.164552.3 (PMC12516180; doi:10.12688/f1000research.164552.3)
Supplement: Peer review history

# Reviewer report: Reviewer response for version 3

**DOI:** 10.5256/f1000research.190310.r428395

**Published:** 2025-11-11

**Authors:** Gaetano Isola

**Affiliations:** 1 University of Catania, Catania, Italy

The manuscript still present the original issues that were not solved. I kindly ask you to give back to my previous comments and answer it.

Is the work clearly and accurately presented and does it cite the current literature?

Partly

If applicable, is the statistical analysis and its interpretation appropriate?

Yes

Are all the source data underlying the results available to ensure full reproducibility?

No

Is the study design appropriate and is the work technically sound?

Partly

Are the conclusions drawn adequately supported by the results?

Partly

Are sufficient details of methods and analysis provided to allow replication by others?

Partly

Reviewer Expertise:

Periodontology

I confirm that I have read this submission and believe that I have an appropriate level of expertise to state that I do not consider it to be of an acceptable scientific standard, for reasons outlined above.

# Reviewer report: Reviewer response for version 2

**DOI:** 10.5256/f1000research.187844.r413621

**Published:** 2025-10-22

**Authors:** Aram Mohammed Sha

**Affiliations:** 1 University of Sulaimani, Sulaimani- Kurdistan Region, Iraq

I have no further comments.

Is the work clearly and accurately presented and does it cite the current literature?

Yes

If applicable, is the statistical analysis and its interpretation appropriate?

Yes

Are all the source data underlying the results available to ensure full reproducibility?

Yes

Is the study design appropriate and is the work technically sound?

Yes

Are the conclusions drawn adequately supported by the results?

Yes

Are sufficient details of methods and analysis provided to allow replication by others?

Yes

Reviewer Expertise:

Periodontology

I confirm that I have read this submission and believe that I have an appropriate level of expertise to confirm that it is of an acceptable scientific standard.

# Reviewer report: Reviewer response for version 2

**DOI:** 10.5256/f1000research.187844.r417608

**Published:** 2025-10-13

**Authors:** Athraa Ali Mahmood

**Affiliations:** 1 Department of Oral Surgery and Periodontics, College of Dentistry, Mustansiriyah University, Baghdad, Baghdad Governorate, Iraq

This study presents interesting and potentially valuable findings. However, some methodological choices are not well-justified, such as the choice of subjects having PPD ≥4 mm, CAL ≥4 mm, and including at least 30% of teeth. Clarify the rationale for methodological decisions, and consider addressing any limitations through additional analysis or discussion. In addition, keywords: arrange the words in alphabetical order, preferably all words in the MeSH. Once these issues are addressed, the manuscript will be a stronger contribution to the field.

Is the work clearly and accurately presented and does it cite the current literature?

Yes

If applicable, is the statistical analysis and its interpretation appropriate?

Yes

Are all the source data underlying the results available to ensure full reproducibility?

Yes

Is the study design appropriate and is the work technically sound?

Partly

Are the conclusions drawn adequately supported by the results?

Yes

Are sufficient details of methods and analysis provided to allow replication by others?

Yes

Reviewer Expertise:

Periodontics

I confirm that I have read this submission and believe that I have an appropriate level of expertise to confirm that it is of an acceptable scientific standard.

# Response

**Published:** 2025-10-24

**Authors:** Shaymaa Abdulkareem Nori

**Affiliations:** University of Baghdad, Baghdad, Baghdad Governorate, Iraq

We thank the reviewer for this valuable feedback. In response, we have clarified the rationale for selecting subjects with PPD ≥4 mm, CAL ≥4 mm, and ≥30% of teeth, referencing the 2018 classification of periodontal diseases (Tonetti et al., 2018) to justify the definition of generalized periodontitis. A new paragraph titled *“Rationale for Methodological Choices”* has been added to the **Methods** section to explain these criteria in detail.

 Additionally, we have expanded the **Discussion** section with an *“Additional Considerations and Limitations”* paragraph to acknowledge the potential effects of inclusion criteria and measurement variability.

 Finally, the **Keywords** have been rearranged alphabetically and standardized according to MeSH terminology.

 We appreciate the reviewer’s insightful comments, which have improved the clarity and rigor of our manuscript.

# Reviewer report: Reviewer response for version 2

**DOI:** 10.5256/f1000research.187844.r417609

**Published:** 2025-10-10

**Authors:** Gaetano Isola

**Affiliations:** 1 University of Catania, Catania, Italy

In the manuscript entitled: “Evaluation of Salivary HLA-DR4 and MMP-8 Levels Along with Porphyromonas gingivalis in Periodontitis Patients with Rheumatoid Arthritis" the author aimed to assess the clinical and immunological characteristics of PD in individuals with and without RA and to compare these characteristics with a healthy control group.

 The author found that salivary HLA-DR4 levels were significantly lower in patient groups compared with healthy controls, whereas salivary MMP-8 and ACPA levels were significantly elevated in

 patients and respectively, with no significant differences between the patient groups. On the other hand, microbial load was significantly higher in the PD with and without RA groups than that in controls, with a positive correlation between microbial load and CAL in the PD-RA group.

 Major comments:

 In general, the idea and innovation of this study regards the analysis of the link between periodontitis and factors associated is interesting and novel because the role these aspects in medicine are validated but further studies on this topic could be an innovative issue in this field could be open a creative matter of debate in literature by adding new information. Moreover, there are few reports in the literature that studied this interesting topic with this kind of study design.

 The study was well conducted by the authors; However, there are some concerns to revise that are described below.

 The introduction section resumes the existing knowledge regarding the important factor linked with the relationship between periodontitis and related mediators and outcomes-

 However, as the importance of the topic, the reviewer strongly recommends, before a further re-evaluation of the manuscript, to update the literature through read, discuss and cites in the references with great attention all of those recent interesting articles, that helps the authors to better introduce and discuss the impact of mirnas in and ecographic vascular outcomes following treatment that could improved periodontitis treatment and in order to obtain a more precise diagnosis by adding these relevant references: [Reference 1], [Reference 2]

 The authors should be better specified, at the end of the introduction section, the rationale of the study and the aim of the study. In the central section, should better clarify inclusions and exclusions criteria of the selected sample.

 Please better state the results obtained in the abstract.

 The discussion section appears well organized with the relevant paper that support the conclusions, even if the authors should better discuss the relationship regarding oxidative stress evolution linked with periodontitis. The conclusion should reinforce in light of the discussions.

 In conclusion, I am sure that the authors are fine clinicians who achieve very nice results with their adopted protocol. However, this study, in my view does not in its current form satisfy a very high scientific requirement for indexing in this journal and requests a revision before a further re-evaluation of the manuscript.

 Minor Comments:

 Abstract:

- Better formulate the abstract section by better describing the aim of the study

Introduction:

- Please refer to major comments

Discussion

- Please add a specific sentence that clarifies the results obtained in the first part of the discussion

Is the work clearly and accurately presented and does it cite the current literature?

Partly

If applicable, is the statistical analysis and its interpretation appropriate?

Yes

Are all the source data underlying the results available to ensure full reproducibility?

No

Is the study design appropriate and is the work technically sound?

Partly

Are the conclusions drawn adequately supported by the results?

Partly

Are sufficient details of methods and analysis provided to allow replication by others?

Partly

Reviewer Expertise:

Periodontology

I confirm that I have read this submission and believe that I have an appropriate level of expertise to confirm that it is of an acceptable scientific standard, however I have significant reservations, as outlined above.

## References

[1] : Impact of periodontal microRNAs associated with alveolar bone remodeling during orthodontic tooth movement: a randomized clinical trial. *Journal of Translational Medicine* .2024;22(1) : 10.1186/s12967-024-05933-x 10.1186/s12967-024-05933-x 39736760 PMC11684316

[2] : Impact of nonsurgical periodontal treatment on arterial stiffness outcomes related to endothelial dysfunction: A systematic review and meta‐analysis. *Journal of Periodontology* .2025;96(4) : 10.1002/JPER.24-0422 330-345 10.1002/JPER.24-0422 PMC1206272739549247

# Reviewer report: Reviewer response for version 2

**DOI:** 10.5256/f1000research.187844.r414214

**Published:** 2025-10-09

**Authors:** Ghasaq Asim Abdul-Wahhab

**Affiliations:** 1 Mustansiriyah University, Baghdad, Baghdad Governorate, Iraq

I have provided a few Specific comments addressed in a manuscript to follow.

 - Abstract: as you use the ROC curve to test the diagnostic potentials of the studied markers; the aim should be targeted to this matter in addition to your first aim.

 - key words: ****use Italic style to write bacterial name.

 - Study population:****

 1. according (WHO, 2000)

 BMI from 18.5-24.9 kg/m ^2 ^considered as normal weight. Specify please

 2. As you followed the recent classification system for periodontal disease; please put the required criteria for periodontitis cases selection.

 - Clinical periodontal parameters: ****The phrase " In contrast, CAL refers to the measurement from cemento-enamel junction (CEJ) to the pocket’s bottom in case of gingival regression" OMITT "in case of gingival regression" as its incorrect.

 - Detection of *Pophyromonas gingivalis* in dental plaque:**** For specific detection and quantification of *P. gingivalis* bacteria, primers were synthesized; please provide the used primer sequence and key characteristics like melting temp.  in single table

 - Statistical analysis: as your data non normally distributed and the non parametric statistical tests were used; there is no need to mention ANOVA and parametric tests.

  Table 2:  For clarity, name the second group (PD without RA) and ensure that all tables  follow the same naming pattern.

 * please provide the statistical tests used in footnotes of each table.

 - Genetic analysis of Pophyromonas gingivalis: use Italic style for bacterial name.

 - Diagnostic accuracy of salivary biomarkers: (PD with and without RA according to ROC data had good accuracy in assessing HLA-DR4, MMP-8 and ACPA sensitivity and specificity ) this phrase should be rephrased as the ROC curve used to test the measured markers in discriminating between patients and controls, it will be: {HLA-DR4, MMP-8 and ACPA according to ROC data had good accuracy in descriminating between PD with and without RA vs controls} and no need for figure 2 as both groups were patients.

 - Spearman correlation test coefficients: no need to mention(microbial load counts) either microbial load or microbial count.

 Discussion:(The diagnostic analysis of salivary biomarkers, including ACPA, HLA-DR4 and MMP-8, using ROC curves comparing PD with RA vs. PD without RA, ............ ) omit this part.

Is the work clearly and accurately presented and does it cite the current literature?

Yes

If applicable, is the statistical analysis and its interpretation appropriate?

Yes

Are all the source data underlying the results available to ensure full reproducibility?

Yes

Is the study design appropriate and is the work technically sound?

Yes

Are the conclusions drawn adequately supported by the results?

Yes

Are sufficient details of methods and analysis provided to allow replication by others?

Yes

Reviewer Expertise:

Periodontology

I confirm that I have read this submission and believe that I have an appropriate level of expertise to confirm that it is of an acceptable scientific standard.

# Response

**Published:** 2025-10-24

**Authors:** Shaymaa Abdulkareem Nori

**Affiliations:** University of Baghdad, Baghdad, Baghdad Governorate, Iraq

We appreciate the reviewer’s thoughtful comments and believe these revisions have substantially strengthened our manuscript in clarity and methodological precision.

# Reviewer report: Reviewer response for version 1

**DOI:** 10.5256/f1000research.181080.r394190

**Published:** 2025-08-08

**Authors:** Ali A Abdulkareem

**Affiliations:** 1 University of Baghdad, Baghdad, Baghdad Governorate, Iraq

The authors aimed to assess the clinical and immunological characteristics of periodontitis in individuals with and without rheumatoid arthritis and to compare these characteristics with a healthy control group. While the efforts of authors are well appreciated; however, several concerns were detected which could affect the internal validity and generalizability of this study.

- The claim of the authors that subjects with pristine gingiva is not well accepted even by the latest classification of periodontal disease which consider those with BOP <10% as healthy periodontium. This means, in real life, bleeding to a limited is accepted as a healthy state of gingiva.
- In connection with the previous comment, no clear case definition of “pristine” or healthy gingiva were stated.
- The study should include a group of RA patients with healthy periodontium to control all confounding factors that may affect the level of salivary biomarkers.
- If the stage of periodontitis was not considered, why the selection was limited to sites with CAL greater or equal to 4mm?
- In clinical periodontal parameters, the authors used unusual terms such as “unrestricted” gingival border!!
- Who measured the clinical parameters? Was the examiner calibrated?
- When collecting subgingival plaque samples from healthy sites, it is not recommended to use curette which is traumatic. Paper points should be used instead especially if the gingiva was healthy or “pristine”.
- Table 1, by which the duration of RA was measures? Days, months, years?
- In the same table, it is not appropriate to express p value as 0.0000. Unique systems of expressing these values are available and should be followed.
- Value of PI that belongs the healthy group is relatively high.
- What is the point of inserting ROC curves without further interpretation? What is the benefit of this analysis from clinical point of view? 

Is the work clearly and accurately presented and does it cite the current literature?

No

If applicable, is the statistical analysis and its interpretation appropriate?

Partly

Are all the source data underlying the results available to ensure full reproducibility?

Partly

Is the study design appropriate and is the work technically sound?

No

Are the conclusions drawn adequately supported by the results?

No

Are sufficient details of methods and analysis provided to allow replication by others?

No

Reviewer Expertise:

Periodontics

I confirm that I have read this submission and believe that I have an appropriate level of expertise to state that I do not consider it to be of an acceptable scientific standard, for reasons outlined above.

# Response

**Published:** 2025-09-06

**Authors:** Shaymaa Abdulkareem Nori

**Affiliations:** University of Baghdad, Baghdad, Baghdad Governorate, Iraq

We thank the reviewer for this valuable suggestions. All revisions have been made as requested by the reviewer and here are some answers for some of the reviewers questions 

 1. we agree that the term pristine gingiva is not consistent with the terminology used in the 2017 world workshop on the classification of periodontal diseases, we used this term to decrease the plagiarism percentage. This term have been changed to healthy gengiva in the updated version 

 2. We fully agree that including a group of RA patients with a clinically healthy periodontium would provide a stronger control against confounding factors. Unfortunately, due to the limited number of RA patients without periodontal involvement attending our clinics during the recruitment period, such a group could not be established.

 3. Our selection of sites with CAL ≥4 mm was intended to ensure that participants included in the periodontitis group presented with a clear diagnosis of periodontitis, in line with standard case definitions. While we did not stratify the cases by stage, the CAL threshold provided consistency in diagnosis. We have clarified this rationale in the Methods section.

 4. The term “unrestricted gingival border” was inadvertently used to describe the “free gingival margin.” The text has been revised accordingly to use the correct terminology.

 5. Clinical parameters were measured by the examiner. First the examiner was trained to apply a precise probing force. Then, periodontal parameters for five patients (not enrollment in this study) were measured for all teeth except third molar by the researcher and the specialist at the same time until an agreement was reached for all clinical periodontal parameters.

 6. We agree that paper points are less traumatic for healthy sites. In our protocol, curettes were used in both groups to maintain uniformity in sample collection. Here is an article they support our method: Wang J, Geng Y, Guo J, Peng J, Xu H, Zhao B, Huang S, Qin M, Du W, Tian J. The Subgingival Microbial Composition in Health and Periodontitis with Different Probing Depths. Microorganisms. 2025 Apr 17;13(4):930. doi: 10.3390/microorganisms13040930. PMID: 40284767; PMCID: PMC12029188.

 7. the duration of RA was recorded in years. This information has been added to table 1

 8. The relatively elevated PI in the healthy group may be attributed to variations in oral hygiene practices among the participants, the control group was not instructed to brush their teeth before the examination to avoid the bias.

 9. ROC curves have been fully interpreted in the revised version.

 best regards 

 Shaymaa A. Nori

 author

# Reviewer report: Reviewer response for version 1

**DOI:** 10.5256/f1000research.181080.r395768

**Published:** 2025-07-28

**Authors:** Aram Mohammed Sha

**Affiliations:** 1 University of Sulaimani, Sulaimani- Kurdistan Region, Iraq

**General comments:**

1. The manuscript needs polishing (grammar, conciseness) by a native proofreader.

**Specific comments:**

**1. Title and Abstract**

**Title:**

- Suggestion: Could specify “salivary levels” earlier to highlight non-invasive diagnostic focus.

**✔** **Abstract:**

 Please consider the following:

- “Periodontitis (PD) is a persistent infectious inflammatory condition” → is periodontitis considered as an infectious? And it is an inflammatory disease or condition.
  - Grammar: “RA, an autoimmune ailment that has been linked with raised periodontal disease severity.” → Incomplete sentence.
  - The sentence “The current findings showed that salivary level of HLA-DR4 was decreased…” lacks clarity and transitions poorly.
  - Missing p-values or effect sizes in the results.

**2. Introduction**

- Some citations are not consistently formatted (e.g., "Mahmood andAl-Ghurabi, 2020" has spacing issues).
- Sentences like: “It has been suggested that upon infection…” are speculative. Preferably cite with “has been demonstrated”.
- Grammar corrections:
  - “RA… presenting considerable obstacles owing to its relentless nature…” → rephrase for clarity.
  - Avoid redundancy, e.g., “shared genetic profile… shared epitope…” — streamline.

**3. Methods**

**Study Design:**

- Sample categorization is somewhat buried; better presented in bullet/table.
- BMI < 25 kg/m² inclusion is restrictive; could have justified it.
- Data handling and storage are sound, but some procedural steps are overly detailed (e.g., pipetting steps could be summarized).
- Include **reference citations** for validated assays (ELISA kits, qRT-PCR protocols).

**4. Sample Collection & Biomarker Measurement**

- Well-explained collection process for **saliva and plaque**.
- ELISA and qPCR methods detailed, but too verbose for a research article — could be shortened and moved to supplementary material.
- The formulas for DAS-28 are useful but might fit better in a methods appendix.

**5. Statistical Analysis**

- Should mention if **normality** was assessed before applying parametric tests.
- Clarify whether tests were two-tailed and if adjustments were made for multiple comparisons.

**6. Results**

- Use of “mean rank” instead of actual values in some tables can obscure clinical relevance.
- ROC curve figures are referenced but not discussed in enough detail.

**7. Discussion**

**Areas for Improvement:**

- Overuse of references in some parts (e.g., entire paragraph on HLA alleles); could be condensed.
- Some repetitive points: ACPA and MMP-8 comparisons repeated several times.
- Claim: “RA exacerbates periodontal inflammation” is supported, but better to qualify it as "in this cohort".
- Better distinction needed between **correlation** and **causation**.

**8. Conclusions**

- Could be more concise.
- Add a sentence on clinical implications, such as the **non-invasive diagnostic potential** of salivary biomarkers.

**9. References**

- Broad and current literature base (many from 2023–2024).
- Formatting is inconsistent:
  - Some entries miss DOIs.
  - Typos: “Ali AA, Khalid KE, Mohammed SE, et al. : Association of Human Ali Leukocyte Antigen…”

Is the work clearly and accurately presented and does it cite the current literature?

Yes

If applicable, is the statistical analysis and its interpretation appropriate?

Yes

Are all the source data underlying the results available to ensure full reproducibility?

Yes

Is the study design appropriate and is the work technically sound?

Yes

Are the conclusions drawn adequately supported by the results?

Yes

Are sufficient details of methods and analysis provided to allow replication by others?

Yes

Reviewer Expertise:

Periodontology

I confirm that I have read this submission and believe that I have an appropriate level of expertise to state that I do not consider it to be of an acceptable scientific standard, for reasons outlined above.

# Response

**Published:** 2025-09-06

**Authors:** Shaymaa Abdulkareem Nori

**Affiliations:** University of Baghdad, Baghdad, Baghdad Governorate, Iraq

We sincerely thank the reviewer for this insightful report. all revisions have been done as requested.

 regarding the reviewer comment  on the use of mean rank, mean rank was used because the data was not normally distributed. other than this, all the changes requested by the reviewer were considered.

 best regards

 Dr. Shaymaa A. Nori

 Author
